# HPV-Related Knowledge and Impact of Patient–Provider Communication on HPV-Associated Cervical Cancer Awareness and Pap Smear Completion in US Women Aged 21–65 Years

**DOI:** 10.3390/cancers17071188

**Published:** 2025-03-31

**Authors:** Adrienne Dean, Nada Eldawy, Jennifer Mendonca, Diana Lobaina, Yasmine Zerrouki, Goodness Okwaraji, Vama Jhumkhawala, Sara Burgoa, Chinenye Lynette Ejezie, Panagiota Kitsantas, Maria Mejia, Lea Sacca

**Affiliations:** 1 Charles E. Schmidt College of Medicine, Florida Atlantic University, Boca Raton, FL 33431, USA; adean2023@health.fau.edu (A.D.); neldawy2022@health.fau.edu (N.E.); jmendonca2016@health.fau.edu (J.M.); dlobaina2021@health.fau.edu (D.L.); yzerrouki2016@health.fau.edu (Y.Z.); gokwaraji2018@health.fau.edu (G.O.); vjhumkhawala2022@health.fau.edu (V.J.); sburgoa2022@health.fau.edu (S.B.); pkitsanta@health.fau.edu (P.K.); mejiam@health.fau.edu (M.M.); 2 Department of Health Sciences, Towson University, Towson, MD 21252, USA; cejezie@towson.edu

**Keywords:** cervical cancer screening, HPV vaccination, Pap smears, patient–provider communication, social determinants of health

## Abstract

Background: The United States (US) continues to face a substantial burden of cervical cancer, which has been the focus of many policies and public health prevention agendas. Of the numerous risk factors associated with cervical cancer, human papillomavirus (HPV) infection remains the leading and most preventable cause of this chronic disease. Therefore, one major public health prevention strategy to decrease cervical cancer cases is HPV vaccination. Another screening tool that enables cervical cancer prevention and early intervention is the Pap smear, the primary method of screening for abnormal cervical cells. However, barriers such as social determinants of health and ineffective patient–provider communication hinder access to such critical preventive measures. The purpose of this study was to provide a comprehensive overview of the knowledge level of US female adults, aged 21–65 years, concerning HPV infection and cervical cancer prevention using the Health Information National Trends Survey (HINTS) database. Additionally, it assessed associations between patient–provider communication and the completion of Pap smear tests. Methods: Descriptive statistics were computed to explore the sociodemographic characteristics of female survey participants as well as to gather frequency and percentages of responses related to knowledge of HPV, awareness of the HPV vaccine, and history of Pap smear. Chi-squared tests were carried out to examine the associations between awareness of a cervical cancer vaccine or HPV shot and whether the participant has had a Pap smear, heard of the HPV vaccine, and knowledge of HPV’s association with cervical cancer. Next, binary logistic regression models were built to determine the size and direction of the association between patient–provider communication metrics and measures of (1) having had a Pap smear, (2) participant knowledge of HPV, (3) participant awareness of causality between HPV and cervical cancer, and (4) participant knowledge of HPV vaccine and cervical cancer prevention measures. Results: A substantial majority of participants (81.8%) reported having heard of HPV. Among them, 72.1% recognized that HPV could cause cervical cancer. Awareness of the HPV vaccine was reported by 88.1%, suggesting a relatively high reach of effective public health messaging. Regarding Pap tests, 43.3% of participants had undergone testing within the past year, but 12.6% had not been tested in over five years, and 3.6% have never been tested. Bivariate analysis using chi-squared tests revealed significant associations between participants’ history of Pap smears and their knowledge of HPV infection, its role in cervical cancer, and HPV vaccination as a prevention tool. Participants who had undergone a Pap test were more likely to have heard of HPV (*p* < 0.001), were knowledgeable of the HPV vaccine (*p* < 0.001), and were more aware of the HPV vaccine (*p* < 0.001). Participants reporting “never” for certain communication criteria significantly had an increased risk of having lower knowledge levels about the HPV vaccine and other cervical cancer prevention measures. They also had almost twice the risk of having lower knowledge levels about HPV prevention measures when reporting “never” (RR = 1.997, 95% CI (1.018–3.916) for “spending enough time with patients” compared to those selecting “always”. Additionally, participants responding “sometimes” (RR = 1.889, 95% CI (1.187–3.005) rather than “always” to feeling involved in healthcare decisions had a significantly higher risk of being unaware of the vaccine or other cervical cancer prevention measures. Conclusions: Strengthening provider communication and education skills not only encourages greater patient knowledge and adherence to preventative measures, such as HPV and cervical cancer screening, but also reduces disparities in healthcare stemming from limited health literacy.

## Simple Summary

Human papillomavirus (HPV) infection remains the leading and most preventable cause of cervical cancer. One major public health prevention strategy to decrease cervical cancer cases is HPV vaccination. Another screening tool that enables cervical cancer prevention and early intervention is the Pap smear, the primary method of screening for abnormal cervical cells. However, barriers related to the individual’s social determinants of health and ineffective patient–provider communication hinder access to such critical preventive measures. Our findings suggest that strengthening provider communication and education skills not only encourages greater patient knowledge and adherence to preventative measures, such as HPV and cervical cancer screening, but also reduces disparities in healthcare stemming from limited health literacy.

## 1. Introduction

Cervical cancer has the eighth-highest incidence of cancer worldwide and is the ninth leading cause of mortality by malignancy [1]. However, among women, cervical cancer is the most diagnosed cancer in 25 countries, second only to breast cancer, and the most common cause of cancer-related death in 37 countries [1]. The United States (US) continues to face a substantial burden of cervical cancer, which has been the focus of many policies and public health prevention agendas [2]. As of 2023, the incidence of cervical cancer in the US is about 11.5 per 100,000 women aged 15–75 years [3].

Although cervical cancer is rare in US women under the age of 30, those aged 50 and below account for more than 50% of the cervical cancer burden, with a disproportionate mortality (70%) in women aged 50 and older [4,5]. Additionally, Black women experience higher incidence rates, estimated at 9.8 per 100,000 for US Black women compared to 8.0 per 100,000 in their White counterparts [3]. Cervical cancer deaths also disproportionately affect Black women, whose mortality rates are twice as high as those of White women [3]. Moreover, Hispanic and Asian/Pacific Islander women also face higher cervical cancer incidence rates compared to their White counterparts, though not as pronounced as the disparity between Black and White women [4]. These racial and ethnic disparities in US cervical cancer morbidity and mortality were further magnified in the Southern states, contributing to regional disparities across the nation [6].

Despite these high rates of cervical cancer-associated mortalities and morbidities, it is important to note that the implementation of effective cervical cancer screening methods over the past 50 years has led to a steady decline in disease incidence, particularly in the US [2,7]. Of the numerous risk factors associated with cervical cancer, including early age of sexual debut, multiple sexual partners, history of sexually transmitted infections, and smoking, human papillomavirus (HPV) infection remains the leading and most preventable cause of this chronic disease [5,7]. Therefore, one major public health prevention strategy to decrease cervical cancer cases is HPV vaccination [8]. The HPV vaccine provides protection against cervical cancer and is routinely recommended for children ages 9–12. Catch-up vaccinations are recommended for individuals under the age of 26, while shared decision-making between patient and provider is recommended by the Centers for Disease Control and Prevention (CDC) for adults older than 26 years as more people in this age group have already been exposed to HPV, offering less benefit from the vaccine [9]. Since the introduction of these vaccines in 2006, there has been an 80.9% decline in HPV infections in women ages 14–19 with continuous slight decreases in incidence rates each year after, thus significantly lowering the greatest risk factor for cervical cancer [10,11,12].

Another screening tool that enables cervical cancer prevention and early intervention is the Pap smear, the primary method of screening for abnormal cervical cells [8,13]. The current recommendation calls for a Pap smear every 3 years between the ages of 21 and 65, balancing specificity and sensitivity while maximizing compliance with screening protocol [13]. Many studies have shown that appropriate follow-up for positive Pap smears can reduce the morbidity and mortality of cervical cancer [14]. Using these screenings to identify women at a higher risk allows for earlier intervention to prevent progression to invasive cervical cancer [13]. However, it is important to note that screenings, diagnostics, and treatments are usually less utilized in minority populations due to limited accessibility [3]. These disparities related to factors such as race/ethnicity, socioeconomic status, education, rurality, and insurance status, also known as social determinants of health, further exacerbate cervical cancer mortality and morbidity rates [10,11,12,13,14].

Social determinants of health (SDoH) refer to the conditions in the environments where people are born, live, learn, work, play, worship, and age which influence a wide range of health, functioning, and quality-of-life outcomes and risks [15]. Cervical cancer screening rates are significantly influenced by various SDoH, as outlined in Healthy People 2030 [15]. Key barriers include socioeconomic factors, such as limited access to healthcare services and lack of health insurance, which can deter women from undergoing regular Pap smear tests [16]. Education level and literacy also play crucial roles as lower educational attainment is often linked with reduced awareness about cervical cancer screening guidelines [17,18]. Additionally, cultural and societal norms can impact participation rates, with certain populations facing stigma or misinformation about the screening process [19,20]. Environmental barriers such as geographical isolation or inadequate transportation infrastructure also hinder access to screening facilities, especially in rural areas [21,22,23]. Furthermore, the availability of healthcare resources, including a shortage of trained healthcare providers and limited clinic hours, can restrict access to timely and appropriate screening services [23]. Each of these barriers underscores the necessity for tailored interventions that address SDoH-related challenges to improve Pap smear completion rates and reduce cervical cancer disparities [24].

Additionally, patient–provider communication stands out as a critical barrier to cervical cancer screening, with the quality of interactions playing a pivotal role in the uptake of regular Pap smears based on the recommended guidelines [25]. Healthcare providers are central to this dynamic, as their recommendations for screenings are highly influential; however, not all providers consistently recommend routine screenings, which can lead to variations in patient compliance [26]. Furthermore, the principle of patient-centered care emphasizes shared decision-making, where patients are not only informed about the benefits and risks of screening but also engaged in discussions that respect their preferences and values [27]. This approach can be complicated by significant barriers, such as language differences and cultural differences between patients and providers. Such barriers can impede effective communication, leading to misunderstandings and potentially lower screening rates among women from diverse backgrounds [28]. Addressing these issues requires targeted training for healthcare providers in cultural competence and communication skills, as well as ensuring the availability of translation services and culturally tailored educational materials to enhance understanding and trust in the screening process [25].

The purpose of this study was to provide a comprehensive overview of the knowledge level of US female adults, aged 21–65 years, concerning HPV infection and cervical cancer prevention using the Health Information National Trends Survey (HINTS) database. Additionally, it assessed associations between patient–provider communication and the completion of Pap smear tests. The findings may shed light on the impact of shared decision-making facilitated by patient–provider communication on preventive behaviors, specifically through the completion of recommended Pap smear tests.

## 2. Methods

### 2.1. Survey Administration and Sample Selection

Data were extracted from the 2022 Health Information National Trends Survey 6 (HINTS 6), a nationally representative survey conducted by the National Cancer Institute [29]. This survey collects information from the American public regarding knowledge of, attitudes toward, and use of cancer- and health-related information, along with data on health-related needs, accessibility, and quality of healthcare received. The survey employed a two-stage design [29]. Initially, a stratified sample of addresses was selected from a database compiled by the Marketing Systems Group, followed by the selection of one adult per household. This iteration of HINTS expanded the traditional sampling strata—previously divided into high and low minority groups—by incorporating a rural/urban stratification to enhance rural representation [29]. Data collection occurred between 7 March 2022 and 8 November 2022. Each selected household was contacted through four mailings: an initial mailing, a reminder postcard, and two follow-up mailings. A subsample of non-respondents received a third follow-up mailing, offering a $30 incentive to complete the survey, aiming to improve response rates. Respondents had the option to respond via paper or through a web survey using a unique access code. All participants received a preliminary $2 pre-paid incentive to encourage survey completion. The survey was available in both English and Spanish.

### 2.2. Analytic Sample

Of the total 6252 participants in HINTS 6, only those who identified as female were included in the analysis for this study (N = 3535). Since the recommended age of screening for cervical cancer is 21–65 years old, ref. [13], our sample was further narrowed to include a total of 2286 women in this age group. Missing data for the demographic variables considered in this study ranged from 1.7% to 7%.

### 2.3. Study Measures

The analytic sample included only women between the ages 21–65 years of age. Age was treated as a continuous variable in the analyses. Occupational status included five categories: employed, homemaker/student/retired/disabled, unemployed, multiple occupation statuses, and other occupations. Marital status was categorized into three groups: married (which included individuals who were married or living as married with a romantic partner), divorced/widowed/separated, and single. Educational level was categorized into less than high school, completed high school/post-high school training, some college, and college graduate/postgraduate. Race included White, Black, Asian (Asian Indian, Chinese, Filipino, Vietnamese, other Asian), multiple races, other, Pacific Islander, and American Indian or Alaska Native. Hispanic origin, which included Mexican, Puerto Rican, Cuban, other Hispanic, and multiple Hispanic ethnicities, was recoded as a binary variable distinguishing Hispanic versus non-Hispanic. Income level was divided into the following ranges: $0 to $49,999; $50,000–$99,999; $100,000–$199,999; and $200,000 or more. The sample characteristics are shown in Table 1.

Regarding knowledge variables, four questions were selected from the HINTS 6 survey related to knowledge of HPV and cervical cancer prevention and detection (Table 2).

Have you ever heard of HPV? HPV stands for Human Papillomavirus. It is not HIV, HSV, or herpes (Yes/No).

Do you think HPV can cause cervical cancer? (Yes/No/Not Sure). This measure was recoded into a binary variable, yes/no, and did not include the not sure category due to a small number of observations.

A vaccine to prevent HPV infection is available and is called the cervical cancer vaccine or HPV shot. Before today, have you ever heard of the cervical cancer vaccine or HPV shot? (Yes/No).

When was your last Pap screen? This measure was recoded into a categorical variable into four categories: 1 = “<= 1 year”; 2 = “1–5 years ago”; 3 = “More than five years ago”; and 4 = “I have never had a Pap test”.

### 2.4. Patient–Provider Communication Variables

Questions were also selected related to participant perception of quality of patient–provider communication (PPC) in their communication with doctors, nurses, or other health professionals in the past 12 months (Table 3). There were seven items related to PPC: (1) how often did they explain things in a way you could understand?; (2) how often did they spend enough time with you?; (3) how often did they involve you in decisions about your health care as much as you wanted?; (4) how often did they give you the chance to ask all the health-related questions you had?; (5) how often did they give the attention you needed to your feelings and emotions?; (6) how often did they make sure you understood the things you needed to do to take care of your health?; and (7) how often did they help you deal with feelings of uncertainty about your health or health care? Measures of PPC quality were answered on a 4-point Likert scale ranging from “Always” to “Never.” All values listed as “Missing date (not ascertained)”, “Missing data (Web partial—question never seen)”, “Multiple responses selected in error”, “Unreadable”, or “Question answered in error (commission error)” were recoded into System Missing and not included in the descriptive tables or statistical analyses.

### 2.5. Statistical Analyses

Descriptive statistics (frequencies and counts) were computed to explore the sociodemographic characteristics of female survey participants (Table 1), as well as to gather the frequency and percentages of responses related to knowledge of HPV, awareness of the HPV vaccine, and history of Pap smear (Table 2). Unweighted frequencies and percentages were also calculated for each of the seven criteria on patient–provider communication for responses on the 4-Likert scale (Table 3). Chi-squared tests were carried out to examine the associations between awareness of a cervical cancer vaccine or HPV shot and whether the participant has had a Pap smear, heard of the HPV vaccine, and knowledge of HPV’s association with cervical cancer (Table 4). Next, binary logistic regression models were built to determine the size and direction of the association between patient–provider communication metrics and measures of (1) having had a Pap smear, (2) participant knowledge of HPV, (3) participant awareness of causality between HPV and cervical cancer, and (4) participant knowledge of HPV vaccine and cervical cancer prevention measures (Table 5). These models were adjusted for sociodemographic characteristics shown in Table 1. Statistical significance was determined at *p* < 0.05. Weights were used to adjust for the complex population-based survey design of HINTS. All data were analyzed using IBM SPSS Statistics (version 29).

## 3. Results

### 3.1. Demographic Characteristics of Participants

The demographic characteristics of the participants are summarized in Table 1. The sample comprised of 2286 females aged 21–65 years (n = 2286). The average age of the sample was around 47 years, with the majority being employed (62.19%), married, or living with a romantic partner (52.53%), and holding a college degree or higher (49.82% college graduates/postgraduates + 20.78% with some college = 70.6%). More than two-fifths (41.39%) reported an annual income below $50,000; most participants identified as non-Hispanic (78.12%) and White (67.48%). These demographic details provide a foundation for understanding the context within which health-related behaviors and knowledge are examined.

### 3.2. Knowledge of HPV and Cervical Cancer Prevention

As depicted in Table 2, a substantial majority of the participants (81.8%) reported having heard of HPV. Among them, 72.1% recognized that HPV could cause cervical cancer. Awareness of the HPV vaccine was reported by 88.1%, suggesting a relatively high reach of effective public health messaging. Regarding Pap tests, 43.3% of the participants had undergone testing within the past year, but 12.6% had not been tested in over five years, and 3.6% have never been tested.

### 3.3. Patient–Provider Communication Quality

Table 3 outlines participants’ perceptions of the quality of their communication with healthcare providers.

Although participants reported positive feedback patient–provider communication on the seven listed criteria, a substantial number of participants (at least 40%) either reported only usual, some, or no instances of their ability to ask questions, feel heard, involved in healthcare decisions, confident in information received, understand doctor, spend enough time with doctors, and deal with feeling of uncertainty.

### 3.4. Bivariate Analysis to Examine Associations Between Pap Smear History and Knowledge of HPV

The bivariate analysis using chi-squared tests revealed significant associations between participants’ history of Pap smears and their knowledge of HPV infection, its role in cervical cancer, and HPV vaccination as a prevention tool. Participants who had undergone a Pap test were more likely to have heard of HPV (*p* < 0.001), were knowledgeable of the HPV vaccine (*p* < 0.001), and were more aware of the HPV vaccine (*p* < 0.001) (Table 4).

### 3.5. Logistic Regression Analysis of Associations Between Patient–Provider Communication and HPV-Related Variables

Logistic regression was carried out to explore the association between the seven criteria of patient–provider communication and whether participants (1) have had a Pap smear; (2) know of HPV; (3) are aware of HPV-associated cervical cancer; and (4) are knowledgeable of the HPV vaccine and cervical cancer prevention measures, while controlling for specific SDoH (sociodemographic characteristics) as covariates.

#### 3.5.1. Associations with Having Had a Pap Smear

Compared to participants who reported “always” having the chance to ask all health-related questions, those who responded “usually” and “sometimes” were 2.536 (1.419–4.533) and 3.164 (1.559–6.419) times more likely, respectively, to report not having a Pap smear (*p* < 0.001). Regarding providers’ attention to participants’ feelings, participants responding “sometimes” had over two times the risk (RR = 2.821, 95% CI (1.494–5.328) of not having a Pap smear compared to those who responded “always”. Similarly, when it comes to involvement in healthcare decisions, participants responding “usually” (RR = 1.996, 95% CI (1.093–3.646) and “sometimes” (RR = 2.827, 95% CI (1.428–5.594) on this criterion had higher risk of not having a Pap smear compared to those responding “always”. Additionally, for the criterion on provider’s help with participants’ feelings of uncertainty about their health, those responding “sometimes” (RR = 2.108, 95% CI (1.106–4.016) instead of “always” were more likely to have no Pap smear history. Finally, those reporting “sometimes” rather than “always” on sufficient time spent with their providers criterion had a significantly higher risk of having no Pap smear history (RR = 3.419, 95% CI (1.755–6.661), suggesting that less-than-optimal provider interaction was associated with lower Pap smear completion rates.

#### 3.5.2. Associations with HPV Knowledge, HPV-Associated Cervical Cancer Risk, and Vaccine Awareness

When exploring the relationship between patient–provider communication criteria and each of HPV knowledge and HPV-associated cervical cancer risk, no statistically significant associations were found. However, those reporting “never” for certain communication criteria significantly had an increased risk of having lower knowledge levels about the HPV vaccine and other cervical cancer prevention measures. For instance, those who responded “never” to having providers explain things in a way they could understand had nearly eight times the risk (RR = 7.997, 95% CI (2.357–27.140) of having low knowledge on HPV vaccination and other cervical cancer prevention measures compared to those reporting “always”.

They also had almost twice the risk of having lower knowledge levels about HPV prevention measures when reporting “never” (RR = 1.997, 95% CI (1.018–3.916) for “spending enough time with patients” compared to those selecting “always”. Additionally, participants responding “sometimes” (RR = 1.889, 95% CI (1.187–3.005) rather than “always” to feeling involved in healthcare decisions had a significantly higher risk of being unaware of the vaccine or other cervical cancer prevention measures. These findings underscore the nuanced impact of patient–provider communication on both screening behaviors and knowledge regarding cervical cancer prevention. Clear and consistent communication appears to be essential for promoting higher Pap smear uptake and a more informed understanding of HPV and its vaccine.

## 4. Discussion

Our study aimed to understand of the levels of knowledge among US adult women aged 21–65 regarding HPV infection and cervical cancer prevention while also assessing the impact of patient–provider communication quality on (1) Pap smear history, (2) knowledge of HPV and HPV-associated cervical cancer, and (3) awareness of HPV vaccination as modalities of cervical cancer prevention.

While 81.8% of survey respondents reported knowledge of HPV, about 20% lacked awareness or did not recognize its oncogenic capabilities. This is concerning, particularly given HPV’s established role in cervical and other anogenital cancers. Although 88.1% of participants were aware of the HPV vaccine, fewer recognized HPV’s direct role in cervical cancer. HPV is detected in 52.5% of atypical squamous cells of undetermined significance (ASCUS) lesions, 74.8% of low-grade cervical lesions, and 88.9% of high-grade cervical lesions [30]. Overall, 4.5% of all cancer cases are attributable to HPV annually, with 8.6% of all cancer cases in women being HPV-related and 0.8% of all cancer cases in men, including head and neck cancers and anogenital cancers [30]. However, despite the documented role of HPV in cervical and other anogenital cancers, many women remain unaware of HPV’s significant contribution to cancer risk [30]. A 2015 anonymous, cross-sectional questionnaire completed by women ages 13–25 revealed that only 41.9% were aware that HPV is associated with cancer [31]. Moreover, while 80% agreed with the importance of Pap smears, only 64.8% knew that these are used to screen for cervical cancer [31]. A similar cross-sectional study in 2018 conducted in community clinics of underserved populations in Texas found that 52% of their sample correctly identified that there is an association between HPV and the development of cervical cancer [32]. In 2022, a short-term quasi-experimental educational intervention in Alabama showed that correct answers to the question “Is cervical cancer caused by HPV infection?” increased from 53% pre-intervention to 91% post-intervention [33]. These studies suggest that while significant gaps in HPV awareness persist, evidence-based educational interventions and patient–provider communication may be well-positioned to bridge the gap.

Our analysis revealed a significant association between the quality of patient–provider interactions and both the knowledge of and engagement with preventive measures. Only 56% of participants reported “always having the chance to ask” health-related questions with their providers, while less than half perceived that their healthcare providers effectively addressed their emotional and health concerns. Participants who reported effective communication were not only more likely to be up to date with Pap tests but also exhibited greater awareness of HPV and the cervical cancer vaccine. This aligns with our bivariate analysis findings showing significant associations between awareness of HPV vaccine and having undergone a Pap test as part of routine gynecological care. Such findings are also supported by established literature which posits that high-caliber communication is instrumental in enhancing patient engagement and adherence to preventive health measures [34,35].

Patient–provider communication is essential to for improving HPV vaccination rates and promoting adherence to recommended Pap smear guidelines [34,35]. However, it has been observed that healthcare providers often lack comprehensive knowledge about HPV vaccination, and their recommendations can be unclear or inconsistent, which may impact adherence [34,35,36,37]. When recommendations for HPV vaccinations are “high-quality”, they demonstrate higher HPV vaccination uptake and lower rates of refusal and delay of adherence [5]. Similarly, there is a lack of emphasis on cervical cancer screening from providers compared to other cancer screenings, like colorectal cancer [38]. It has also been demonstrated that those providers who perceive patients to have lower health literacy or presume knowledge of the patient’s level of education place patients at risk for inadequate follow-up for an abnormal Pap smear [39]. Interventions that incorporate direct communication versus virtual or telephone contact can increase compliance with cervical cancer screening [40]. Additionally, education can help providers deliver effective recommendations to improve adherence to HPV and Pap smear guidelines [34,35,41,42,43,44,45].

Furthermore, our sample was predominantly White (67.48%) and non-Hispanic (78.12%). Studies have found that HPV vaccination initiation, vaccine completion rates, as well as cervical cancer screening and follow-up are lower among Hispanic, Black, Asian, and American Indian/Alaskan Natives [37,38,44]. Additionally, ethnic minority women in the US are more likely to receive a diagnosis of cervical cancer and more likely to receive a diagnosis of cervical cancer at more advanced stages, leading to increased mortality rates among these populations [45,46,47,48,49]. Racial and ethnic minority adults also report higher trust in educators and healthcare providers from similar backgrounds [36]. Therefore, enhancing diversity among health educators and providers, tailoring information to promote understanding of a given population, and providing high-quality patient–provider communication are crucial in improving health literacy and supporting ethnic minorities, underserved, and immigrant populations [36].

Significant associations were found between patient–provider communication criteria and completion of Pap smears, knowledge of HPV, participant awareness of HPV-associated cervical cancer, and knowledge of HPV preventive measures. The findings suggest that responses such as “sometimes”, or “never” in relation to positive experiences with the provider increased the risk of not having had a Pap smear, a lack of knowledge about HPV, limited awareness of the connection between HPV and cervical cancer, and insufficient knowledge of the HPV vaccine and cervical cancer prevention measures. Previous studies have shown how SDoH, such as age, race/ethnicity, education, transportation, financial stability, access to care or transportation, and insurance status, play a role in influencing patient–provider communication and healthcare decision-making, particularly regarding timely completion of HPV vaccination and gynecological visits [38,44,49,50,51,52]. Moss et al. (2016) demonstrated that although collaborative communication between providers and patients is positively associated with HPV vaccination compliance, underserved individuals tend to experience less collaborative communication with providers, which leads to lower vaccination compliance [49]. However, vaccination rates increase when individuals completed educational programs in their language or if the study controlled for communication across demographic groups [49]. Mansfield et al. 2022 showed that among Hispanic families, language barriers along with cultural stigmatizations created challenges when communicating with providers; for instance, one individual mentioned misunderstanding the initial HPV vaccination conversation with their provider, such that “there was a second [vaccination]” [50]. Additionally, even with initiatives in place that target disadvantaged populations to make healthcare more acceptable, like the Affordable Care Act or the CDC’s National Breast and Cervical Cancer Early Detection Program, those who are uninsured or who have Medicaid insurance continue to be under-screened for cervical cancer [44,53,54].

The advent of regular cervical screening and HPV vaccination has led to clear public health benefits. The American Cancer Society reports that cervical cancer incidence rates decreased by more than 50% from the 1970s until the mid-2000s, coinciding with the beginning of regular cervical cancer screening in outpatient offices [55]. Furthermore, rates of cervical cancer declined 11% each year from 2012–2019 for women between the ages 20 to 24, as HPV vaccination rates increased within this population. Providing information regarding screening and intervention for cervical cancer in various forms to target a wider audience, especially across diverse ethnic, racial, and socioeconomic groups, could increase the impact of HPV vaccination and cervical screenings [55]. Education tailored to specific demographics, through consideration of the nuances between cultures and groups, can make screening and vaccination more effective. Examples of this exist in the literature. A study by Roh and Lee found that educational tools tailored to American Indian women, a group disproportionately affected by breast cancer, regarding breast cancer screening promoted screening behaviors and increased overall knowledge [56]. Increasing screening and vaccination rates could lead to even greater cancer reduction outcomes nationally.

Expanding upon this finding, broader health policies that encourage and facilitate communication and cultural humility-based training would establish effective communication as a core component of medical education [57]. By integrating these concepts throughout training, providers can apply these skills in practice, ultimately delivering more tailored care to patients [57,58]. A larger pool of physicians with these skills would enhance the quality of care and help mitigate existing disparities among underserved populations. A study by Warren et al. found that implementing educational tools such as information kiosks at health centers increased HPV vaccine uptake amongst low-income women and increased their knowledge levels about cervical cancer prevention [58]. Physicians who are equipped with information about HPV vaccination and cervical screening can consistently provide this information to a broad range of patients and, furthermore, tailor their communication to individual patient needs.

Finally, there has been a call for action from the World Health Organization to implement a global strategy to accelerate the elimination of cervical cancer as a public health problem [59]. This strategy proposes a target of 90-70-90 that must be met by 2030 for countries to be on the path towards cervical cancer and consists of the following goals: (1) 90% of girls be fully vaccinated with HPV vaccine by age of 15, (2) 70% of women be screened with a high performance test by 35 years of age and again at the age of 45, and (3) 90% of women identified with cervical disease receive treatment [59]. Widespread coverage of both HPV vaccination and cervical screening from 2020 onwards has the potential to prevent up to 12·5–13·4 million cervical cancer cases by 2069 [60]. Hence, ongoing global discussions on how to improve cervical cancer prevention measures that take into consideration the role of providers and the effectiveness of patient–provider communication are essential to enhance elimination targets for this chronic disease [60].

## 5. Limitations

This study has various limitations due to its cross-sectional design. The data for this study were derived from the HINTS 6 survey, which was completed through an in-person or random digital-dial mixed-methods interview. Random digital dial calls can lead to limited response rates among the study population, as well as diminished validity of certain measures at baseline. Another limitation is the sample demographics. The majority of the sample consisted of White and non-Hispanic women, which may have influenced the results concerning patient–provider communication and provided less insight into demographic differences in responses. We also limited our sample to women aged 21–65 years old as this is the recommended age group for screening; however, this decreased the number of participants in our sample. Furthermore, social desirability bias may have been introduced by participants reporting higher levels of comfort in communicating with their providers. The cross-sectional nature of the HINTS study prevents the establishment of causality between the role of patient–provider communication and HPV knowledge, vaccination, and cervical cancer awareness rates. Another limitation affecting the data collection process was the COVID-19 pandemic. Since the survey was collected post-pandemic in 2022, declining cervical cancer screening rates might also be related to the overall impact of the pandemic on screening behaviors. A study by Star et al. showed that cervical cancer screening rates remained below pre-pandemic levels in 2023, a troubling trend as early-stage diagnoses continued to decrease in 2021 [61]. The study also emphasized that the persistent decline may in part reflect longer-term declines in patient knowledge and clinician recommendation of cervical cancer screening [61]. Therefore, additional efforts are needed to combat the negative consequences of the pandemic on preventive behaviors. Despite these limitations, this study is one of the few that compare the significance of associations between patient-centered, informed decision-making for HPV vaccination and cervical cancer screening alongside the influential role of SDoH. Future research should look deeper into the effects of SDoH within ethnic minorities and low-income populations on decision-making regarding HPV vaccination and cervical cancer prevention. Eliciting data helps establish causality between certain communication methods and increased health literacy would be useful in determining which types of educational approaches are most effective for providing information to different patient groups. In addition, exploring implementation science frameworks that tailor in-person interactions with healthcare providers could help make them more effective as well as increase overall health literacy.

## 6. Conclusions

Cervical cancer education and prevention rely on effective communication between healthcare providers and patients. Providing screening information to patients in an approachable, clear, and relevant manner fosters understanding and allows for shared decision-making regarding their cervical health. Giving at-risk females tailored information not only allows them to understand their options for cervical cancer screening and HPV vaccination but also informs them about the risks of forgoing preventative measures. Larger scale health policies encouraging the development of communication skills within medical education programs would allow for even more successful preventative health campaigns. Furthermore, educating future providers to communicate with a variety of populations using culturally relevant tools will aid in mitigating disparities in healthcare by making this information accessible to all. Overall, strengthening provider communication and education skills not only encourages greater patient knowledge and adherence to preventative measures, such as HPV and cervical cancer screening, but also reduces disparities in healthcare stemming from limited health literacy.

## Figures and Tables

**Table 1:** Sociodemographic Characteristics of Adult Female Participants Aged 21–65 years old.

Demographic Characteristics	N (%) Unweighted n (Weighted %)
Age (n = 2286)	
21–65	2286 (100%), Mean = 47.12 (Standard deviation= 12.5)
Occupational Status (n = 2264)	
Employed	1408 (62.19%)
Homemaker/student/retired/disabled	342 (15.10%)
Unemployed	282 (12.46%)
Multiple occupation statuses	180 (7.95%)
Other occupation	52 (2.30%)
Marital Status (n = 2252)	
Married/living as married or living with a romantic partner	1183 (52.53%)
Divorced/widowed/separated	544 (24.16%)
Single	525 (23.31%)
Education (n = 2194)	
Less than high school	128 (5.84%)
Completed high school/post-high school training	517 (23.56%)
Some college	456 (20.78%)
College graduate/postgraduate	1093 (49.82%)
Hispanic Origin (n = 2198)	
Not Hispanic	1717 (78.12%)
Hispanic	481 (21.88%)
Race (n = 2082)	
White	1405 (67.48%)
Black	421 (20.22%)
Asian	108 (5.19%)
Multiple races selected	93 (4.47%)
Other Pacific Islander	33 (1.59%)
American Indian or Alaska Native	22 (1.05%)
Income Level (n = 2126)	
$0 to $49,999	880 (41.39%)
$50,000 to $99,999	638 (30.01%)
$100,000 to $199,999	428 (20.13%)
$200,000 or more	180 (8.47%)

**Table 2:** Knowledge of HPV and HPV-Associated Cervical Cancer, HPV Vaccination, and Pap Smear History.

Have you heard of HPV? (N = 2019)	
Yes	1651 (81.77%)
No	368 (18.23%)
Do you think HPV can cause cervical cancer? (N = 1636)	
Yes	1179 (72.07%)
No	457 (27.93%)
Before today, have you heard of cervical cancer vaccine or HPV shot? (N = 1634)	
Yes	1439 (88.07%)
No	195 (11.93%)
When was your last Pap screen? (N = 1615)	
≤1 year	699 (43.28%)
1–5 years ago	654 (40.50%)
More than 5 years ago	204 (12.63%)
I have never had a Pap test	58 (3.59%)

**Table 3:** Patient–Provider Communication Criteria.

*Give you the chance to ask all healthcare-related questions*			
Always	Usually	Sometimes	Never
823 (56%)	487 (33%)	143 (10%)	15 (1%)
*Give the attention you needed to your feelings and emotions*			
Always	Usually	Sometimes	Never
615 (42%)	545 (37%)	249 (17%)	59 (4%)
*Involve you in decisions about your health care as much as you wanted*			
Always	Usually	Sometimes	Never
772 (53%)	484 (33%)	187 (13%)	25 (2%)
*Make sure you understood the things you needed to do to take care of your health*			
Always	Usually	Sometimes	Never
803 (55%)	486 (33%)	159 (11%)	20 (1%)
*Explain things in a way you could understand*			
Always	Usually	Sometimes	Never
860 (59%)	488 (33%)	108 (7%)	12 (1%)
*Spend enough time with you*			
Always	Usually	Sometimes	Never
577 (39%)	517 (35%)	308 (21%)	66 (5%)
*Help you deal with feelings of uncertainty about your health or healthcare*			
Always	Usually	Sometimes	Never
550 (38%)	512 (35%)	314 (21%)	92 (6%)

**Table 4:** Bivariate Associations between Knowledge of HPV, Cervical Cancer Prevention, and Pap Smear History.

Have you ever heard of HPV?			
Pap Smear History	Yes	No	*p*-value
Had Pap Test	1731 (77.73%)	396 (17.78%)	<0.001 *
Never Had Pap Test	62 (2.78%)	38 (1.71%)	
Before today, have you ever heard of the cervical cancer vaccine or HPV shot?			
Pap Smear History	Yes	No	*p*-value
Had Pap Test	1218 (68.66%)	494 (27.85%)	<0.001 *
Never Had Pap Test	28 (1.58%)	34 (1.91%)	
Do you think HPV can cause cervical cancer?			
Pap Smear History	Yes	No	*p*-value
Had Pap Test	1506 (84.89%)	206 (11.61%)	0.004 *
Never Had Pap Test	47 (2.65%)	15 (0.85%)	

* Significance set at *p* < 0.05.

**Table 5:** Logistic Regression of the Association between Patient–Provider Communication Criteria with Not Having a Pap Smear, Lack of Knowledge Regarding HPV, Lack of Awareness of the Causality between HPV and Cervical Cancer; and Lack of Knowledge of HPV Vaccine and Cervical Cancer Prevention Measures. * All of the logistic regression models were adjusted for the sample characteristics shown in Table 1. ** Significance set at *p* < 0.05.

Patient–Provider Communication Criteria	Have Not Had a Pap Smear *			Lack of Knowledge Regarding HPV *			Lack of Awareness of the Causality Between HPV and Cervical Cancer *			Lack of knowledge about the HPV Vaccine and Cervical Cancer Prevention Measures *		
	RR	95% CI	Sig. **	RR	95% CI	Sig.	RR	95% CI	Sig.	RR	95% CI	Sig.
Chance to ask all health-related questions												
Always	Ref	Ref	Ref	Ref	Ref	Ref	Ref	Ref	Ref	Ref	Ref	Ref
Usually	2.536	1.419–4.533	<0.001 **	0.858	0.643–1.146	0.300	1.186	0.911–1.545	0.205	0.903	0.611–1.335	0.609
Sometimes	3.164	1.559–6.419	<0.001 **	1.030	0.680–1.560	0.888	1.048	0.691–1.591305	0.824	1.415	0.839–2.387	0.193
Never	5.180	0.623–6.873	0.984	1.766	0.702–4.447	0.227	0.253	0.0508457–1.256438	0.093	1.369	0.341–5.499	0.658
Provide attention needed to patient’s feelings and emotions												
Always	Ref	Ref	Ref	Ref	Ref	Ref	Ref	Ref	Ref	Ref	Ref	Ref
Usually	1.395	0.739–2.635	0.305	0.762	0.565–1.028	0.075	1.021	0.778–1.340	0.881	0.964	0.647–1.437	0.858
Sometimes	2.821	1.494–5.328	<0.001 **	1.170	0.836–1.638	0.359	0.854	0.601–1.214	0.380	1.17	0.729–1.878	0.515
Never	0.350	0.044–2.758	0.319	0.778	0.388–1.560	0.480	0.724	0.369–1.419	0.346	1.509	0.687–3.314	0.305
Allow involvement in healthcare decisions to patient’s satisfaction												
Always	Ref	Ref	Ref	Ref	Ref	Ref	Ref	Ref	Ref	Ref	Ref	Ref
Usually	1.996	1.093–3.646	0.024 **	1.006	0.754–1.343	0.965	0.963	0.733–1.267	0.790	1.056	0.706–1.578	0.792
Sometimes	2.827	1.428–5.594	0.003 **	1.108	0.763–1.608	0.591	1.284	0.894–1.845	0.176	1.889	1.187–3.005	0.007 **
Never	1.543	0.324–7.342	0.585	1.115	0.458–2.719	0.810	0.755	0.284–2.007	0.573	1.267	0.403–3.982	0.686
Ensure patient understanding about actions to be taken to improve health												
Always	Ref	Ref	Ref	Ref	Ref	Ref	Ref	Ref	Ref	Ref	Ref	Ref
Usually	1.361	0.762–2.433	0.298	0.896	0.674–1.191	0.450	1.095	0.838–1.431	0.507	0.757	0.503–1.140	0.183
Sometimes	1.847	0.913–3.735	0.088	0.777	0.503–1.200	0.255	1.188	0.802–1.761	0.390	1.561	0.954–2.555	0.076
Never	0.694	0.087–5.563	0.731	1.472	0.617–3.514	0.384	0.747	0.255–2.189	0.594	2.023	0.680–6.015	1.27
Explain things to ensure patient understanding												
Always	Ref	Ref	Ref	Ref	Ref	Ref	Ref	Ref	Ref	Ref	Ref	Ref
Usually	1.103	0.614–1.980	0.743	0.945	0.711–1.255	0.694	1.061	0.814–1.383	0.663	0.843	0.564–1.261	0.405
Sometimes	2.0138	0.976–4.153	0.058	1.257	0.815–1.937	0.301	1.414	0.908–2.201	0.126	1.952	1.150–3.312	0.013 *
Never	1.467	0.180–12.009	0.719	1.521	0.502–4.604	0.458	1.127	0.312–4.070	0.855	7.997	2.357–27.140	<0.001 **
Spend enough time with patient												
Always	Ref	Ref	Ref	Ref	Ref	Ref	Ref	Ref	Ref	Ref	Ref	Ref
Usually	1.537	0.770–3.070	0.223	0.931	0.691–1.255	0.640	0.950	0.715–1.262	0.723	0.789	0.514–1.212	0.279
Sometimes	3.419	1.755–6.661	<0.001 **	0.988	0.704–1.387	0.945	1.198	0.867–1.654	0.274	1.320	0.846–2.059	0.221
Never	1.554	0.485–4.986	0.458	0.887	0.481–1.636	0.700	0.798	0.432–1.476	0.473	1.997	1.018–3.916	0.044 **
Help patient deal with feelings of uncertainty about their health												
Always	Ref	Ref	Ref	Ref	Ref	Ref	Ref	Ref	Ref	Ref	Ref	Ref
Usually	1.048	0.541–2.031	0.890	0.987	0.735–1.326	0.933	0.979	0.735–1.304	0.885	0.915	0.599–1.399	0.683
Sometimes	2.108	1.106–4.016	0.023 **	0.798	0.556–1.139	0.214	1.08	0.781–1.496	0.640	1.243	0.792–1.951	0.345
Never	0.990	0.322–3.043	0.986	0.749	0.422–1.327	0.321	0.768	0.447–1.320	0.339	1.444	0.746–2.794	0.276

## Data Availability

The authors used data from a national public dataset, the Health Information National Trends Survey (HINTS) database, and can share the specific datasets used upon request.

## References

[1] Bray F., Laversanne M., Sung H., Ferlay J., Siegel R.L., Soerjomataram I., Jemal A. (2024). Global cancer statistics 2022: GLOBOCAN estimates of incidence and mortality worldwide for 36 cancers in 185 countries. CA A Cancer J. Clin..

[2] Collins Y., Holcomb K., Chapman-Davis E., Khabele D., Farley J.H. (2014). Gynecologic cancer disparities: A report from the Health Disparities Taskforce of the Society of Gynecologic Oncology. Gynecol. Oncol..

[3] Perkins R.B., Wentzensen N., Guido R.S., Schiffman M. (2023). Cervical cancer screening: A review. JAMA.

[4] Watson M., Saraiya M., Benard V., Coughlin S.S., Flowers L., Cokkinides V., Schwenn M., Huang Y., Giuliano A. (2008). Burden of cervical cancer in the United States, 1998–2003. Cancer.

[5] Cohen P.A., Jhingran A., Oaknin A., Denny L. (2019). Cervical cancer. Lancet.

[6] Horner M.J., Altekruse S.F., Zou Z., Wideroff L., Katki H.A., Stinchcomb D.G. (2011). US geographic distribution of prevaccine era cervical cancer screening, incidence, stage, and mortality. Cancer Epidemiol. Biomark. Prev..

[7] Fuzzell L.N., Perkins R.B., Christy S.M., Lake P.W., Vadaparampil S.T. (2021). Cervical cancer screening in the United States: Challenges and potential solutions for underscreened groups. Prev. Med..

[8] Saslow D., Ba K.S.A., Manassaram-Baptiste D., Smith R.A., Fontham E.T., American Cancer Society Guideline Development Group (2020). Human papillomavirus vaccination 2020 guideline update: American Cancer Society guideline adaptation. CA A Cancer J. Clin..

[9] Petrosky E., Bocchini J.A., Hariri S., Chesson H., Curtis C.R., Saraiya M., Unger E.R., Markowitz L.E., Centers for Disease Control and Prevention (CDC) (2015). Use of 9-valent human papillomavirus (HPV) vaccine: Updated HPV vaccination recommendations of the advisory committee on immunization practices. MMWR Morb. Mortal Wkly. Rep..

[10] Kamolratanakul S., Pitisuttithum P. (2021). Human papillomavirus vaccine efficacy and effectiveness against cancer. Vaccines.

[11] Ellingson M.K., Sheikha H., Nyhan K., Oliveira C.R., Niccolai L.M. (2023). Human papillomavirus vaccine effectiveness by age at vaccination: A systematic review. Hum. Vaccines Immunother..

[12] Markowitz L.E., Naleway A.L., Klein N.P., Lewis R.M., Crane B., Querec T.D., Hsiao A., Aukes L., Timbol J., Weinmann S. (2019). Human papillomavirus vaccine effectiveness against HPV infection: Evaluation of one, two, and three doses. J. Infect. Dis..

[13] Liverani C.A., Di Giuseppe J., Giannella L., Delli Carpini G., Ciavattini A. (2020). Cervical cancer screening guidelines in the postvaccination era: Review of the literature. J. Oncol..

[14] Guzick D.S. (1978). Efficacy of screening for cervical cancer: A review. Am. J. Public Health.

[15] Office of Disease Prevention and Health Promotion Social Determinants of Health. Healthy People 2030. https://health.gov/healthypeople/priority-areas/social-determinants-health.

[16] Le H., Ziogas A., Lipkin S.M., Zell J.A. (2008). Effects of socioeconomic status and treatment disparities in colorectal cancer survival. Cancer Epidemiol. Biomark. Prev..

[17] Damiani G., Basso D., Acampora A., Bianchi C.B., Silvestrini G., Frisicale E.M., Sassi F., Ricciardi W. (2015). The impact of level of education on adherence to breast and cervical cancer screening: Evidence from a systematic review and meta-analysis. Prev. Med..

[18] Kim K., Han H.-R. (2016). Potential links between health literacy and cervical cancer screening behaviors: A systematic review: Potential links between health literacy and cervical cancer screening. Psycho-Oncology.

[19] Lee M.C. (2000). Knowledge, barriers, and motivators related to cervical cancer screening among Korean-American women: A focus group approach. Cancer Nurs..

[20] Milner G.E., McNally R.J. (2020). Nonadherence to breast and cervical cancer screening among sexual minority women: Do stigma-related psychological barriers play a role?. Health Psychol..

[21] Lunsford N.B., Ragan K., Smith J.L., Saraiya M., Aketch M. (2017). Environmental and psychosocial barriers to and benefits of cervical cancer screening in Kenya. Oncol..

[22] Daley E., Alio A., Anstey E.H., Chandler R., Dyer K., Helmy H. (2011). Examining barriers to cervical cancer screening and treatment in Florida through a socio-ecological lens. J. Community Health.

[23] Sherris J., Wittet S., Kleine A., Sellors J., Luciani S., Sankaranarayanan R., Barone M.A. (2009). Evidence-based, alternative cervical cancer screening approaches in low-resource settings. Int. Perspect. Sex. Reprod. Health.

[24] Rees I., Jones D., Chen H., Macleod U. (2018). Interventions to improve the uptake of cervical cancer screening among lower socioeconomic groups: A systematic review. Prev. Med..

[25] Agénor M., Bailey Z., Krieger N., Austin S.B., Gottlieb B.R. (2015). Exploring the cervical cancer screening experiences of black lesbian, bisexual, and queer women: The role of patient-provider communication. Women Health.

[26] Han J., Jungsuwadee P., Abraham O., Ko D. (2018). Shared decision-making and women’s adherence to breast and cervical cancer screenings. Int. J. Environ. Res. Public Health.

[27] Jacobs E.A., Karavolos K., Rathouz P.J., Ferris T.G., Powell L.H. (2005). Limited English proficiency and breast and cervical cancer screening in a multiethnic population. Am. J. Public Health.

[28] Genoff M.C., Zaballa A., Gany F., Gonzalez J., Ramirez J., Jewell S.T., Diamond L.C. (2016). Navigating language barriers: A systematic review of patient navigators’ impact on cancer screening for limited English proficient patients. J. Gen. Intern. Med..

[29] National Cancer Institute (NCI) (n.d.) Health Information National Trends Survey. About HINTS. https://hints.cancer.gov/about-hints/learn-more-about-hints.aspx.

[30] Serrano B., Brotons M., Bosch F.X., Bruni L. (2018). Epidemiology and burden of HPV-related disease. Best Pract. Res. Clin. Obstet. Gynaecol..

[31] Ahken S., Fleming N., Dumont T., Black A. (2015). HPV awareness in higher-risk young women: The need for a targeted HPV catch-up vaccination program. J. Obstet. Gynaecol. Can..

[32] Alafifi R., Kindratt T.B., Pagels P., Saleh N., Gimpel N.E. (2019). Awareness and knowledge of human papilloma virus and cervical cancer in women with high pap uptake. J. Community Health.

[33] Banks K.S., James C.M., Nganwa D., Heath J., Webb L., Elhussin I., Faraj R., Abdalla E. (2022). Knowledge and awareness about cervical cancer and Human Papillomavirus among women living in Macon County, Alabama. J. Healthc. Sci. Humanit..

[34] Gilkey M.B., Calo W.A., Moss J.L., Shah P.D., Marciniak M.W., Brewer N.T. (2016). Provider communication and HPV vaccination: The impact of recommendation quality. Vaccine.

[35] Meadows R.J., Gehr A.W., Lu Y., Maynard G., Akpan I.N., Taskin T., Fulda K.G., Patel D., Matches S., Ojha R.P. (2024). Effectiveness of provider communication training for increasing human papillomavirus vaccine initiation at a safety-net health system. Prev. Med. Rep..

[36] Amboree T.L., Darkoh C. (2021). Barriers to human papillomavirus vaccine uptake among racial/ethnic minorities: A systematic review. J. Racial Ethn. Health Disparities.

[37] Xu M.A., Choi J., Capasso A., DiClemente R. (2023). Patient–provider health communication strategies: Enhancing HPV vaccine uptake among adolescents of color. Healthcare.

[38] Peterson E.B., Ostroff J.S., DuHamel K.N., D’Agostino T.A., Hernandez M., Canzona M.R., Bylund C.L. (2016). Impact of provider-patient communication on cancer screening adherence: A systematic review. Prev. Med..

[39] Lindau S.T., Basu A., Leitsch S.A. (2006). Health literacy as a predictor of follow-up after an abnormal Pap smear: A prospective study. J. Gen. Intern. Med..

[40] McDermott A.K., McDermott A.J., Osbaldiston R., Lennon R.P. (2021). Improving breast and cervical cancer screening compliance through direct physician contact in a military treatment facility: A non-randomized pilot study. Mil. Med..

[41] Gilkey M.B., McRee A.-L. (2016). Provider communication about HPV vaccination: A systematic review. Hum. Vaccines Immunother..

[42] Leung S.O.A., Akinwunmi B., Elias K.M., Feldman S. (2019). Educating healthcare providers to increase Human Papillomavirus (HPV) vaccination rates: A Qualitative Systematic Review. Vaccine X.

[43] Spencer J.C., Kim J.J., Tiro J.A., Feldman S.J., Kobrin S.C., Skinner C.S., Wang L., McCarthy A.M., Atlas S.J., Pruitt S.L. (2023). Racial and ethnic disparities in cervical cancer screening from three U.S. healthcare settings. Am. J. Prev. Med..

[44] Holt H.K., Peterson C.E., David S.M., Abdelaziz A., Sawaya G.F., Guadamuz J.S., Calip G.S. (2023). Mediation of racial and ethnic inequities in the diagnosis of advanced-stage cervical cancer by insurance status. JAMA Netw. Open.

[45] Islami F., Fedewa S.A., Jemal A. (2019). Trends in cervical cancer incidence rates by age, race/ethnicity, histological subtype, and stage at diagnosis in the United States. Prev. Med..

[46] Yoo W., Kim S., Huh W.K., Dilley S., Coughlin S.S., Partridge E.E., Chung Y., Dicks V., Lee J.-K., Bae S. (2017). Recent trends in racial and regional disparities in cervical cancer incidence and mortality in United States. PLoS ONE.

[47] Beavis A.L., Gravitt P.E., Rositch A.F. (2017). Hysterectomy-corrected cervical cancer mortality rates reveal a larger racial disparity in the United States. Cancer.

[48] Cohen C.M., Wentzensen N., Castle P.E., Schiffman M., Zuna R., Arend R.C., Clarke M.A. (2023). Racial and ethnic disparities in cervical cancer incidence, survival, and mortality by histologic subtype. J. Clin. Oncol..

[49] Moss J.L., Gilkey M.B., Rimer B.K., Brewer N.T. (2016). Disparities in collaborative patient-provider communication about human papillomavirus (HPV) vaccination. Hum. Vaccines Immunother..

[50] Mansfield L.N., Chung R.J., Silva S.G., Merwin E.I., Gonzalez-Guarda R.M. (2022). Social determinants of human papillomavirus vaccine series completion among U.S. adolescents: A mixed-methods study. SSM Popul. Health.

[51] Shin M.B., Sloan K.E., Martinez B., Soto C., Baezconde-Garbanati L., Unger J.B., Kast W.M., Cockburn M., Tsui J. (2023). Examining multilevel influences on parental HPV vaccine hesitancy among multiethnic communities in Los Angeles: A qualitative analysis. BMC Public Health.

[52] Maness S.B., Thompson E.L. (2019). Social Determinants of human papillomavirus vaccine uptake: An assessment of publicly available data. Public Health Rep..

[53] CDC About the National Breast and Cervical Cancer Early Detection Program. National Breast and Cervical Cancer Early Detection Program. 19 September 2024. https://www.cdc.gov/breast-cervical-cancer-screening/about/index.html.

[54] Hhs.gov. https://www.hhs.gov/healthcare/about-the-aca/index.html.

[55] Siegel R.L., Giaquinto A.N., Jemal A. (2024). Cancer statistics, 2024. CA A Cancer J. Clin..

[56] Roh S., Lee Y.-S. (2023). Developing culturally tailored mobile web app education to promote breast cancer screening: Knowledge, barriers, and needs among American Indian women. J. Cancer Educ..

[57] de Grubb M.C.M., Kilbourne B., Zoorob R., Gonzalez S., Mkanta W., Levine R. (2015). Resident Physicians and Cancer Health Disparities: A Survey of Attitudes, Knowledge, and Practice. J. Cancer Educ..

[58] Warren J.R., Hopfer S., Fields E.J., Natarajan S., Belue R., McKee F.X., Hecht M., Lebed J.P. (2023). Digital HPV education to increase vaccine uptake among low income women. PEC Innov..

[59] WHO (2018). WHO Director-General Calls for All Countries to Take Action to Help End the Suffering Caused by Cervical Cancer.

[60] Simms K.T., Steinberg J., Caruana M., Smith M.A., Lew J.-B., Soerjomataram I., Castle P.E., Bray F., Canfell K. (2019). Impact of scaled up human papillomavirus vaccination and cervical screening and the potential for global elimination of cervical cancer in 181 countries, 2020–2099: A modelling study. Lancet Oncol..

[61] Star J., Han X., Smith R.A., Schafer E.J., Jemal A., Bandi P. (2025). Cancer Screening 3 Years After the Onset of the COVID-19 Pandemic. Payment.

